# Drive Time and Receipt of Guideline-Recommended Screening, Diagnosis, and Treatment

**DOI:** 10.1001/jamanetworkopen.2022.40290

**Published:** 2022-11-04

**Authors:** Arianne K. Baldomero, Ken M. Kunisaki, Chris H. Wendt, Ann Bangerter, Susan J. Diem, Kristine E. Ensrud, David B. Nelson, Carrie Henning-Smith, Bradley A. Bart, Patrick Hammett, Hildi J. Hagedorn, R. Adams Dudley

**Affiliations:** 1 Pulmonary, Allergy, Critical Care, and Sleep Medicine, Minneapolis VA Health Care System, Minneapolis, Minnesota; 2 Pulmonary, Allergy, Critical Care, and Sleep Medicine, University of Minnesota, Minneapolis; 3 Center for Care Delivery and Outcomes Research, Minneapolis VA Health Care System, Minneapolis, Minnesota; 4 General Internal Medicine, Minneapolis VA Health Care System, Minneapolis, Minnesota; 5 Division of Biostatistics, University of Minnesota, Minneapolis; 6 Division of Health Policy and Management, University of Minnesota, Minneapolis; 7 Cardiology, Minneapolis VA Health Care System, Minneapolis, Minnesota

## Abstract

**Importance:**

Many patients do not receive recommended services. Drive time to health care services may affect receipt of guideline-recommended care, but this has not been comprehensively studied.

**Objective:**

To assess associations between drive time to care and receipt of guideline-recommended screening, diagnosis, and treatment interventions.

**Design, Setting, and Participants:**

This cohort study used administrative data from the National Veterans Health Administration (VA) data merged with Medicare data. Eligible participants were patients using VA services between January 2016 and December 2019. Women ages 65 years or older without underlying bone disease were assessed for osteoporosis screening. Patients with new diagnosis of chronic obstructive pulmonary disease (COPD) indicated by at least 2 encounter codes for COPD or at least 1 COPD-related hospitalization were assessed for receipt of diagnostic spirometry. Patients hospitalized for ischemic heart disease were assessed for cardiac rehabilitation treatment.

**Exposures:**

Drive time from each patient’s residential address to the closest VA facility where the service was available, measured using geocoded addresses.

**Main Outcomes and Measures:**

Binary outcome at the patient level for receipt of osteoporosis screening, spirometry, and cardiac rehabilitation. Multivariable logistic regression models were used to assess associations between drive time and receipt of services.

**Results:**

Of 110 780 eligible women analyzed, 36 431 (32.9%) had osteoporosis screening (mean [SD] age, 66.7 [5.4] years; 19 422 [17.5%] Black, 63 403 [57.2%] White). Of 281 130 patients with new COPD diagnosis, 145 249 (51.7%) had spirometry (mean [SD] age, 68.2 [11.5] years; 268 999 [95.7%] men; 37 834 [13.5%] Black, 217 608 [77.4%] White). Of 73 146 patients hospitalized for ischemic heart disease, 11 171 (15.3%) had cardiac rehabilitation (mean [SD] age, 70.0 [10.8] years; 71 217 [97.4%] men; 15 213 [20.8%] Black, 52 144 [71.3%] White). The odds of receiving recommended services declined as drive times increased. Compared with patients with a drive time of 30 minutes or less, patients with a drive time of 61 to 90 minutes had lower odds of receiving osteoporosis screening (adjusted odds ratio [aOR], 0.90; 95% CI, 0.86-0.95) and spirometry (aOR, 0.90; 95% CI, 0.88-0.92) while patients with a drive time of 91 to 120 minutes had lower odds of receiving cardiac rehabilitation (aOR, 0.80; 95% CI, 0.74-0.87). Results were similar in analyses restricted to urban patients or patients whose primary care clinic was in a tertiary care center.

**Conclusions and Relevance:**

In this retrospective cohort study, longer drive time was associated with less frequent receipt of guideline-recommended services across multiple components of care. To improve quality of care and health outcomes, health systems and clinicians should adopt strategies to mitigate travel burden, even for urban patients.

## Key Points

**Question:**

Is drive time associated with receipt of guideline-recommended screening, diagnosis, and treatment?

**Findings:**

In this cohort study evaluating osteoporosis screening among women aged 65 years or older, spirometry to confirm COPD diagnosis, and cardiac rehabilitation after hospitalization for ischemic heart disease, the odds of receiving recommended services significantly declined with longer drive times.

**Meaning:**

These results suggest that patients with longer drive times are less likely to receive guideline-recommended health care services.

## Teaser

This cohort study of US adults receiving care with Veterans Administration services compares the association of drive time with receiving recommended services.

## Introduction

Access to health care services profoundly affects health and well-being. One dimension of health care access is geography, which includes the time required to travel to facilities offering services.^1^ Drive time to health care services may affect receipt of guideline-recommended care, but this has not been comprehensively studied. Prior studies of the associations between drive time and receipt of recommended services in cancer care have had mixed results,^2,3,4,5,6^ but the association of drive time with receipt of recommended services in other specialties is unknown.

The Veterans Health Administration (VA) has instituted system-wide measures that may potentially mitigate how longer drive times may factor into patient care. These include expanding clinics into rural areas, offering telehealth options, allowing patients to receive care from local non-VA facilities, and providing transportation services, travel reimbursement, and overnight stays on VA property. However, it is unknown whether these measures have been sufficient to eliminate the association of greater travel requirements with lower receipt of services. If drive time is associated with receipt of care among veterans, it may also be a factor for patients in other health care systems that have fewer programs that mitigate travel-related barriers to care.

To address the gaps in the literature, we used a national VA data set merged with Medicare data to estimate the association of drive time with receipt of guideline-recommended services. To address generalizability of our findings, we studied 3 process measures that addressed different components of care (prevention, diagnosis, and treatment) and 3 different conditions (osteoporosis, chronic obstructive pulmonary disease [COPD], and ischemic heart disease [IHD]). Although longer drive times can be associated with rural residence, we hypothesized that drive time could also be a barrier for some urban patients or patients who have access to these services in their primary care sites. Therefore, we also assessed the association of drive time with receipt of services in analyses restricted to urban patients and restricted to patients whose primary care was located in a tertiary care facility.

## Methods

This study was approved by institutional review boards at the Minneapolis VA Health Care System and the University of Minnesota, and the requirement for informed consent was waived as this research involved no more than minimal risk of harm to participants. This study followed the Strengthening the Reporting of Observational Studies in Epidemiology (STROBE) reporting guideline for cohort studies.

### Selection of Evidence-Based Process Measures

To select process measures that addressed different components of care and different specialties, we solicited ideas from a physician panel. Panel members included 2 primary care physicians (S.J.D., K.E.E.), 2 pulmonologists (C.H.W., K.M.K.), and a cardiologist (B.A.B.). Selection of the process measures was based on guidelines, taking into consideration the strength of recommendation and level of evidence. The panel recommended a screening measure from primary care (osteoporosis screening), a diagnostic test from pulmonary (confirmation of COPD by spirometry), and a therapeutic intervention from cardiology (cardiac rehabilitation after hospitalization for IHD).

The osteoporosis screening measure was defined as receipt of bone mineral density (BMD) measurements for women ages 65 years or older per the US Preventive Service Task Force (USPSTF) (grade B recommendation; level of certainty, moderate).^7^ The COPD diagnostic measure was defined as receipt of spirometry to confirm airflow obstruction in patients with newly diagnosed COPD, per guidelines from the American College of Physicians, American College of Chest Physicians, American Thoracic Society, and European Respiratory Society (strong recommendation; moderate-quality evidence).^8^ The cardiac therapeutic measure was defined as receipt of cardiac rehabilitation for patients hospitalized after acute myocardial infarction (MI), percutaneous coronary intervention (PCI), or coronary artery bypass grafting (CABG) per guidelines from the American College of Cardiology Foundation and American Heart Association (class I recommendation; level of evidence, B).^9^

### Data Sources

We extracted electronic health record data from the national VA Corporate Data Warehouse (CDW). Services received in the VA were identified from the VA outpatient files. To capture services paid for by the VA but received outside of a VA facility (ie, VA-purchased care), we used the non-VA Care Program integrity tools and fee basis files. Because over 90% of veterans ages 65 years or older are enrolled in Medicare,^10^ we also acquired Medicare outpatient and carrier files to capture services from Medicare fee-for-service.

### Study Populations and Outcomes

**Figure 1.**
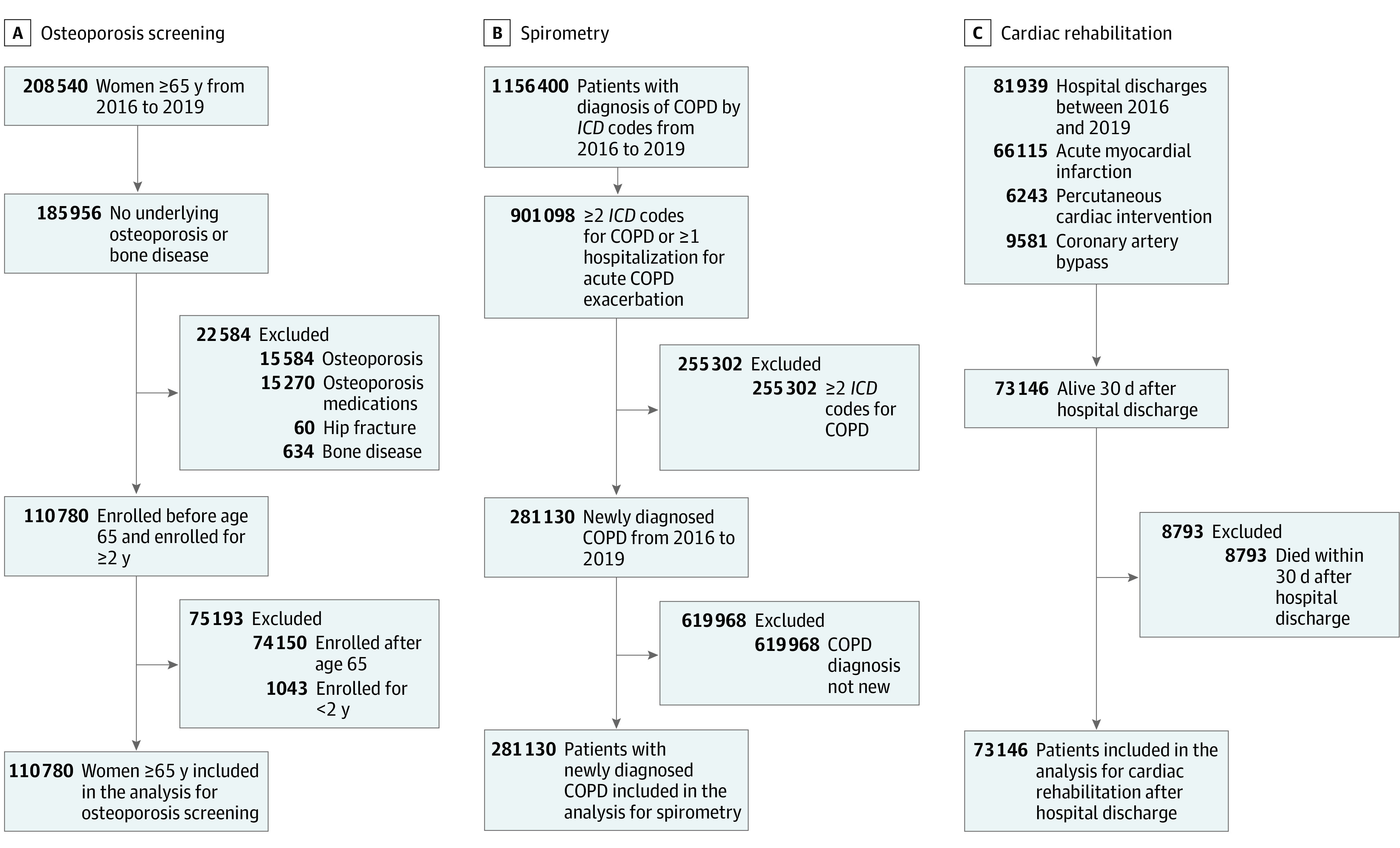
Flow Diagrams for Identification of Patients Eligible for Osteoporosis Screening, Spirometry, and Cardiac Rehabilitation COPD indicates chronic obstructive pulmonary disease; *ICD*, *International Classification of Diseases*.

We identified patients eligible for each of the recommended services from among all individuals using VA services between January 2016 and December 2019 (Figure 1). Diagnosis and procedure codes used are in eTable 1 in the Supplement. We used eligibility criteria and outcome definitions following approaches from other administrative databased studies for each recommended service.^11,12,13,14^ Analyses for spirometry and cardiac rehabilitation included men and women, while osteoporosis screening included women only.

#### Osteoporosis Screening

For osteoporosis screening, we selected women who were aged 65 years or older between 2016 and 2019.^7^ Because we were interested in screening for primary prevention, we excluded patients with preexisting osteoporosis, hip fracture, or underlying conditions that affect bone health as described by Gillespie et al.^11^ To reduce the number of women with BMD measurements not reported in our data, we included women who began VA enrollment before turning 65 and were enrolled for at least 2 years. We defined the index date as the 65th birthday. We identified patients who had BMD measurement by dual-energy x-ray absorptiometry or quantitative ultrasound defined by *Current Procedural Terminology* (*CPT*) and Healthcare Common Procedure Coding System (HCPCS) codes within 2 years after the index date.^11^

#### Spirometry

We included patients with new diagnosis of COPD if they had at least 2 visits with *International Statistical Classification of Diseases and Related Health Problems, Tenth Revision *(*ICD-10*) codes for COPD or at least 1 COPD-related hospitalization between 2016 and 2019.^12^ Since patients who had COPD before our measurement period may have had spirometry that we could not capture in the database, we excluded patients with any diagnosis of COPD before 2016. Among the remaining patients, we identified those who had spirometry, as defined by *CPT* or Pulmonary Function Test clinic stop code (stop code 104), within 2 years before or after the initial COPD diagnosis.^12^

#### Cardiac Rehabilitation

We identified patients hospitalized for acute MI, PCI, or CABG at a VA hospital using *ICD-10*, *CPT*, and HCPCS codes during the study period.^13,14^ We excluded patients who died within 30 days of discharge. Among the remaining patients, we identified those who had cardiac rehabilitation defined by at least 1 *CPT*, HCPCS, or cardiopulmonary rehabilitation clinic stop code (stop code 231) within 12 months of the initial hospital discharge.^13^

### Explanatory Variables

We collected demographic characteristics, geographical location, and comorbid conditions from the CDW. We obtained geocoded patient residential addresses and VA facility addresses, urban and rural designations, and drive times from the VA Planning Systems Support Group Geocoded Enrollee Files.^15^ Drive time estimates from each patient’s residential address to the closest VA facility where the service was available were calculated using geospatial technologies as described by the VA Health Economics Resource Center^16^ and were based on expected driving routes, traffic, and average driving conditions.^15^ The VA designates each patient’s residential address as urban or rural using Rural-Urban Commuting Area (RUCA) codes, version 2010.^17^ We defined urban as RUCA codes 1.0 or 1.1 and rural as all others. This definition of urban is narrow, incorporating only census tracts with a metropolitan area core, but we used these VA urban and rural designations for consistency with VA policy and other research.^15,18^ We quantified socioeconomic disadvantage using the Area Deprivation Index (ADI), which provides percentile ranking of neighborhoods by census block groups based on the aggregated domains of income, education, employment, and housing quality.^19^ We used the Charlson Comorbidity Index (CCI) to assess health status.^20^ Tertiary care designation is assigned to VA facilities with advanced specialized services which include, but are not limited to, cardiac surgery, neurosurgery, or organ transplant.^21^

### Statistical Analysis

We examined the characteristics of patients by the services for which they were eligible. We calculated the percentage of patients who received the recommended service (a binary outcome at the patient level). We used multivariable logistic regression models to estimate the associations between drive time from the patient’s residential address to the closest VA facility where the service was available (categorized into 30 and below [referent group], 31 to 60, 61 to 90, 91 to 120 and over 120 minutes) and receipt of service. We split the drive time into categories based on nonlinear patterns of plots from empirical logit and logistic model parameter estimates of receipt of services by 10-minute drive time increments. Thirty-minute increments resulted in a reasonable approximation of the drive time categories by visual inspection. In our primary analysis, models were adjusted for age, sex, race, urban or rural address, CCI, and ADI to minimize the potential confounding effects of sociodemographic factors and underlying comorbidities. Given that access to and use of health care services vary by race and ethnicity, we included this data to provide an insight into potential differences in receipt of health care services by race and ethnicity. Data were self-reported, and categories included American Indian or Alaska Native, Asian, Black, Native Hawaiian or Pacific Islander, White, and unknown or declined. We used an omnibus likelihood-ratio χ^2^ test to assess whether drive time was associated with receipt of services. We also used likelihood ratio tests to assess whether there was a linear component to the pattern of logs odds of receipt of services by drive time. *P* values <.05 were considered statistically significant in 1-sided tests.

To assess the association of drive time with receipt of services among urban patients and patients who have access to these services in their primary care sites, we performed sensitivity analyses limited to urban patients and to patients with primary care located in tertiary care level VA facilities. We also performed sensitivity analyses restricted to patients aged 65 years or older and enrolled in Medicare fee-for-service during the ascertainment period and analyses excluding patients enrolled in Medicare Advantage (MA) during the ascertainment period. All statistical analyses were performed using SAS software, version 9.4 (SAS Institute).

## Results

Patients included in the analysis for cardiac rehabilitation were slightly older than patients in the analyses for osteoporosis screening and spirometry; patients in the spirometry and cardiac rehabilitation were predominantly men (eTable 2 in the Supplement). Of 110 780 women aged 65 years or older, 36 431 (32.9%) had osteoporosis screening within 2 years of their 65th birthday (mean [SD] age, 66.7 [5.4] years; 19 422 [17.5%] Black, 63 403 [57.2%] White). Of 281 130 patients with a new COPD diagnosis, 145 249 (51.7%) had confirmatory spirometry (mean [SD] age, 68.2 [11.5] years; 268 999 [95.7%] men; 37 834 [13.5%] Black, 217 608 [77.4%] White). Of 73 146 patients hospitalized for ischemic heart disease, 11 171 (15.3%) had cardiac rehabilitation (mean [SD] age, 70.0 [10.8] years; 71 217 [97.4%] men; 15 213 [20.8%] Black, 52 144 [71.3%] White). Rural patients had worse mean ADIs and had more than twice as long median drive times to the closest services than their urban counterparts (eTable 3 in the Supplement). For all 3 recommended services, most (between 56.9% and 70.7%) patients resided in urban areas and had drive times within 30 minutes (Table 1; eTable 4, eTable 5, and eTable 6 in the Supplement).

**Table 1.** Characteristics of Patients at Baseline by Recommended Service, Stratified by Receipt of the Recommended Service

Receipt of recommended service	Patients, No. (%)					
	Osteoporosis screening (n = 110 780)		Spirometry (n = 281 130)		Cardiac rehabilitation (n = 73 146)	
	Yes	No	Yes	No	Yes	No
No.	36 431	74 349	145 249	135 881	11 171	61 975
Age, mean (SD), y	68.3 (5.7)	65.9 (5.1)	68.0 (10.7)	68.4 (12.4)	67.2 (8.8)	70.5 (11.0)
Sex						
Women	36 431 (100)	74 349 (100)	6153 (4.2)	5978 (4.4)	233 (2.1)	1696 (2.7)
Male	NA	NA	139 096 (95.8)	129 903 (95.6)	10 938 (97.9)	60 279 (97.3)
Race						
American Indian or Alaska Native	381 (1.1)	800 (1.1)	1430 (1.0)	1537 (1.1)	106 (1.0)	570 (0.9)
Asian	238 (0.7)	434 (0.6)	620 (0.4)	770 (0.6)	79 (0.7)	410 (0.7)
Black	6096 (16.7)	13 326 (17.9)	18 938 (13.0)	18 896 (13.9)	1432 (12.8)	13 781 (22.2)
Native Hawaiian or Pacific Islander	360 (1.0)	605 (0.8)	1125 (0.8)	1009 (0.7)	83 (0.7)	497 (0.8)
White	23 268 (63.9)	40 135 (54.0)	114 296 (78.7)	103 312 (76.0)	8893 (79.6)	43 251 (69.8)
Unknown or declined	6089 (16.7)	19 049 (25.6)	8840 (6.1)	10 357 (7.6)	578 (5.2)	3466 (5.6)
Area Deprivation Index, mean (SD), percentile^a^	54.1 (25.0)	54.4 (25.6)	58.0 (24.8)	59.0 (24.8)	56.8 (24.6)	58.1 (26.2)
Charlson Comorbidity Index^b^						
0	28 687 (78.7)	61 429 (82.6)	39 301 (27.1)	48 263 (35.5)	2033 (18.2)	9696 (15.7)
1-2	6408 (17.6)	10 209 (13.7)	69 436 (47.8)	56 549 (41.6)	6145 (55.0)	28 610 (46.2)
≥3	1336 (3.7)	2711 (3.7)	36 512 (25.1)	31 069 (22.9)	2993 (26.8)	23 669 (38.2)
Geographic region^c^						
Midwest	5186 (15.5)	9889 (14.6)	31 737 (23.9)	27 217 (21.7)	3131 (30.7)	10 685 (18.7)
Northeast	4074 (12.2)	8354 (12.3)	20 405 (15.4)	18 227 (14.5)	1093 (10.7)	7661 (13.4)
South	14 699 (43.9)	30 470 (45.0)	54 353 (40.9)	51 388 (41.0)	3493 (34.3)	25 002 (43.7)
West	9500 (28.4)	18 978 (28.0)	26 423 (19.9)	28 492 (22.7)	2458 (24.2)	13 854 (24.2)
Rurality						
Urban	24 961 (68.5)	50 960 (68.5)	87 862 (60.5)	81 938 (60.3)	7051 (63.1)	42 674 (68.9)
Rural	11 470 (31.5)	23 389 (31.5)	57 387 (39.5)	53 943 (39.7)	4120 (36.9)	19 301 (31.1)
Drive time to the closest service, min						
≤30	14 306 (39.3)	35 071 (47.2)	76 983 (53.0)	70 565 (51.9)	4976 (44.5)	29 443 (47.5)
31-60	8510 (23.4)	16 827 (22.6)	38 955 (26.8)	34 783 (25.6)	2804 (25.1)	13 308 (21.5)
61-90	4612 (12.7)	8841 (11.9)	16 000 (11.0)	16 142 (11.9)	1303 (11.7)	6415 (10.4)
91-120	3653 (10.0)	6031 (8.1)	7814 (5.4)	8324 (6.1)	892 (8.0)	5461 (8.8)
>120	5350 (14.7)	7579 (10.2)	5497 (3.8)	6067 (4.5)	1196 (10.7)	29 443 (11.9)

Abbreviation: NA, not applicable.

^a^ Area Deprivation Index provides percentile ranking of neighborhoods by census block groups based on the aggregated domains of income, education, employment, and housing quality (percentile ranged from 1 to 100, with higher scores indicating higher levels of socioeconomic disadvantage).^19^

^b^ Charlson Comorbidity Index scores range from 0 to 33, with higher scores indicating greater disease burden and increased risk of death within 1 year.^20^

^c^ Geographic regions were divided into 4 categories according to each patient’s Veterans Integrated Services Networks (VISN) which are regional systems of care working together to meet local health care needs and provides access to care. Midwest includes patients from VISNs 10, 15, 17, and 23; Northeast from VISNs 1, 2, and 4; South from VISNs 6, 7, 8, 9, 16, and 17; and West from VISNs 19, 20, 21, and 22. Numbers may not sum to group totals as some patients were assigned to networks outside of the specified VISNs.

Patients with longer drive times had lower odds of receiving the recommended services compared with patients with shorter drive times (Table 2). For example, compared with patients with a drive time of 30 minutes or less, patients with a drive time of 61 to 90 minutes had lower odds of receiving osteoporosis screening (adjusted odds ratio [aOR], 0.9; 95% CI, 0.86-0.95) and spirometry (aOR, 0.90; 95% CI, 0.88-0.92) while patients with a drive time of 91 to 120 minutes had lower odds of receiving cardiac rehabilitation (aOR, 0.80; 95% CI, 0.74-0.87). The tests for linear trends highlight that spirometry steadily decreased from a drive time of 31 to 60 minutes (aOR, 1.01; 95% CI, 0.99-1.03 vs over 120 min: aOR, 0.82; 95% CI, 0.79-0.86; *P* < .001) and cardiac rehabilitation steadily decreased from a drive time of 61 to 90 minutes (aOR, 0.98; 95% CI, 0.91-1.05 vs over 120 min: aOR, 0.77; 95% CI, 0.72-0.83; *P* < .001); no linear trend was observed for osteoporosis screening (*P* = .31 for linear trend) (Table 2).

**Table 2.** Logistic Regression Analyses for Receipt of Recommended Services by Drive Time to the Closest Service^a^

Drive time	Patients, No.	Adjusted (95% CI)		*P* value for linear trend
		Rates, %	OR	
**Osteoporosis screening (110 780 patients)**				
≤30 min	49 377	35.0 (34.5-35.5)	1 [Reference]	.31
31-60 min	25 337	34.5 (33.9-35.1)	0.98 (0.94-1.01)	
61-90 min	13 453	32.8 (31.9-33.6)	0.90 (0.86-0.95)	
91-120 min	9684	33.4 (32.4-34.4)	0.93 (0.88-0.98)	
>120 min	12 929	33.7 (32.8-34.6)	0.94 (0.90-0.99)	
**Spirometry (281 130 patients)**				
≤30 min	147 548	52.4 (52.1-52.7)	1 [Reference]	<.001
31-60 min	73 738	52.7 (52.3-53.0)	1.01 (0.99-1.03)	
61-90 min	32 142	49.7 (49.2-50.3)	0.90 (0.88-0.92)	
91-120 min	16 138	48.5 (47.7-49.3)	0.86 (0.83-0.89)	
>120 min	11 564	47.5 (46.6-48.5)	0.82 (0.79-0.86)	
**Cardiac rehabilitation (73 146 patients)**				
≤30 min	34 419	14.7 (14.3-15.1)	1 [Reference]	<.001
31-60 min	16 112	15.3 (14.7-15.9)	1.05 (0.99-1.11)	
61-90 min	7718	14.4 (13.6-15.2)	0.98 (0.91-1.05)	
91-120 min	6353	12.2 (11.4-13.0)	0.80 (0.74-0.87)	
>120 min	8544	11.8 (11.1-12.5)	0.77 (0.72-0.83)	

Abbreviation: OR, odds ratio.

^a^ Models adjusted for age, sex, race, urban or rural address, Charlson Comorbidity Index, and Area Deprivation Index. The omnibus likelihood-ratio χ^2^ test to assess whether drive time was associated with receipt of services is *P* < .001 for all models.

In sensitivity analyses restricted to urban patients and patients whose primary care clinic was located in a tertiary care facility, drive time remained significantly associated with receipt of the 3 services (eg, cardiac rehabilitation: 31 to 60 minutes, 1.10; 95% CI, 1.03-1.17 vs over 60 minutes, 0.84; 95% CI, 0.78-0.90) (Table 3). Similarly, drive time remained significantly associated with receipt of services in sensitivity analyses restricted to patients aged 65 years or older and enrolled in Medicare fee-for-service (eTable 7 in the Supplement) and in analyses excluding patients enrolled in MA (eTable 8 in the Supplement).

**Table 3.** Logistic Regression Analyses for Receipt of Recommended Services by Drive Time to the Closest Service Among Patients Residing in Urban Areas and Patients Whose Primary Care Clinics Are Located in a Tertiary Care Facility^a^

Characteristics	Osteoporosis screening		Spirometry			Cardiac rehabilitation		
	Rates, % (95% CI)	Odds ratio (95% CI)	Rates, % (95% CI)		Odds ratio (95% CI)	Rates, % (95% CI)	Odds ratio (95% CI)	
**Patients residing in urban areas**								
Total, No.	75 921		169 800			49 725		
Drive time to the closest service, min								
≤30	34.8 (34.2-35.3)	1 [Reference]	52.4 (52.1-52.7)	1 [Reference]		13.5 (13.1-13.9)		1 [Reference]
31-60	34.2 (33.4-35.0)	0.976 (0.937-1.016)	52.0 (51.5-52.6)	0.986 (0.962-1.010)		14.6 (13.9-15.3)		1.099 (1.028-1.173)
>60	33.3 (32.5-34.1)	0.937 (0.897-0.979)	47.6 (46.8-48.4)	0.824 (0.797-0.853)		11.5 (10.9-12.2)		0.836 (0.779-0.896)
**Patients whose primary care clinic is located in a tertiary care facility^b^**								
Total, No.	34 429		53 627			29 075		
Drive time to the closest service, min								
≤60	33.7 (33.1-34.4)	1 [Reference]	52.9 (52.5-53.4)	1 [Reference]		14.6 (14.2-15.1)		1 [Reference]
>60	31.1 (29.8-32.5)	0.888 (0.826-0.953)	47.9 (46.5-49.3)	0.817 (0.770-0.868)		14.3 (13.2-15.4)		0.899 (0.810-0.996)

^a^ All models were adjusted for age, sex, race, Charlson Comorbidity Index, and Area Deprivation Index. The model for patients whose primary care clinics are located in a tertiary care center was also adjusted for urban/rural address. The omnibus likelihood-ratio χ^2^ test to assess whether drive time was associated with receipt of services is *P* < .001 for all models.

^b^ Tertiary care designation is assigned to VA facilities with advanced specialized services, which include, but are not limited to, cardiac surgery, neurosurgery, or organ transplantation.^21^ All patients whose primary care is located in a tertiary care facility had access to osteoporosis screening and spirometry, while 92.3% of these patients had access to cardiac rehabilitation.

**Figure 2.**
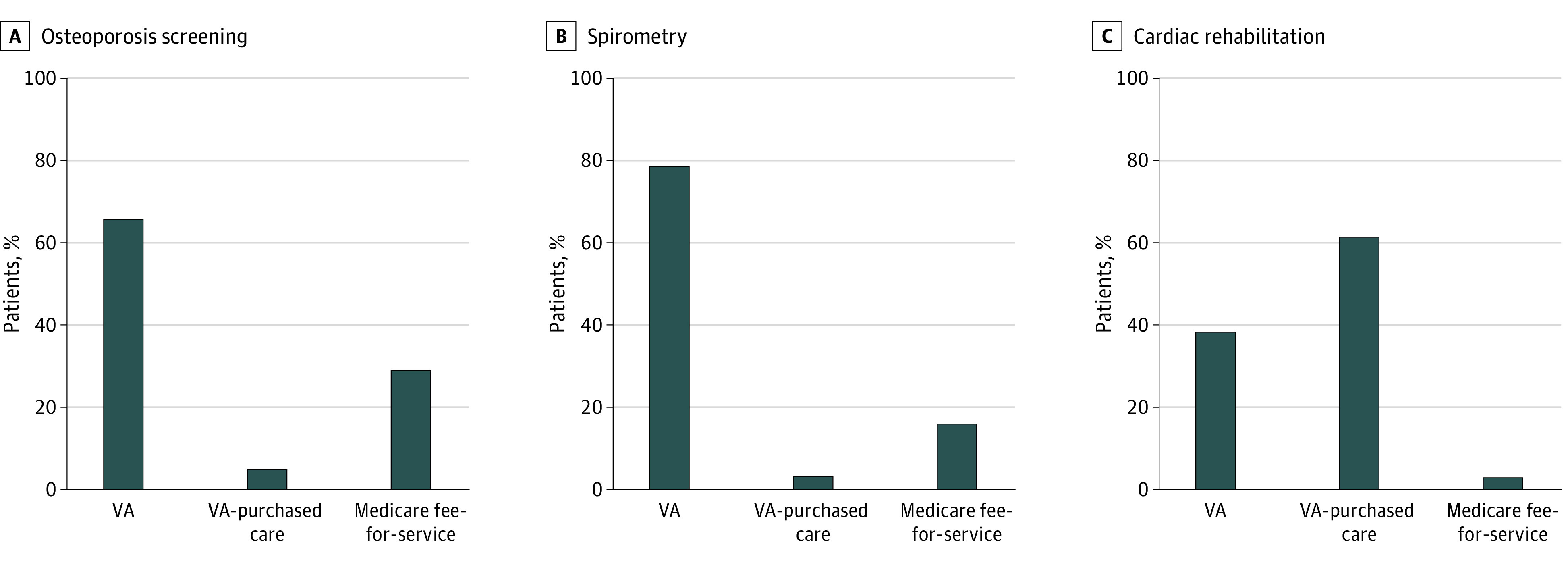
Proportion of Patients Who Received the Recommended Health Service in a VA or non-VA facility Non-VA facility services included VA-purchased care and Medicare fee-for-service.

Most osteoporosis screening and spirometry were completed in a VA facility (24 008 [65.9%] and 115 763 [79.7%], respectively), while most cardiac rehabilitation (7015 [62.8%]) was provided through VA-purchased care. Medicare fee-for-service accounted for 10 711 osteoporosis screening events (29.4%), 24 547 spirometry tests (16.9%), and 369 cardiac rehabilitation services (3.3%) (Figure 2).

## Discussion

Longer drive time was associated with less frequent receipt of guideline-recommended services. This association was observed across care provided by different medical specialties and across screening, diagnostic, and therapeutic care. These associations persisted among urban patients and in patients receiving primary care located in tertiary care centers, suggesting that drive time may be relevant even to patients not traditionally thought to face geographic and transportation barriers to care.

Our data show that, for all 3 services, rural patients had median drive times over twice as long as their urban counterparts. This suggests that future studies evaluating rural-urban disparities should incorporate drive time as a possible mechanism by which disparities might arise.

We also found that drive time is a barrier for urban patients and patients whose primary care is located within tertiary care facilities. These findings suggest that even for clinicians and clinical institutions based in urban areas—and even using the VA’s narrow definition of urban—drive time may be an important, and perhaps, underrecognized barrier to access to care.

Prior studies report mixed results about the association of drive time with receipt of care. However, these studies have narrowly focused on cancer care and often been from a single center, state, or province.^2,3,4,5,6^ Several studies addressed drive distance, also mostly in cancer care.^22,23,24,25,26,27,28^ Compared with drive time, drive distance may less accurately capture the burden of travel because some distances require longer travel than others, especially in dense urban areas.^29,30^

Of note, the overall rates of receiving guideline-recommended services were low in our study, but this is consistent with prior studies performed in both VA and non-VA patients. Among MA and commercial enrollees, 26.5% of women ages 65 to 79 years old were screened for osteoporosis over 2 years.^11^ For confirmatory spirometry among patients with COPD, the reported rates are approximately 30% regardless of insurance type in the US,^31,32^ although rates are reported to be higher in Canada (41.2% to 56%).^33,34^ Cardiac rehabilitation rates among patients on Medicare are between 16.3% and 24.4%, while rates within the VA have previously been reported to be between 8.7% and 10.3%.^13,35,36^ Multiple factors at the patient, clinician, and health system levels likely contribute to low receipt of these services, but we found that drive time was consistently associated with receipt of recommended care.

The magnitude of differences in adjusted rates for receipt of services between the shortest and the longest drive times are modest (Table 2). The reduction in receipt of services between drive times below 30 minutes and over 120 minutes was 1.3% for osteoporosis screening, 4.9% for spirometry, and 2.9% for cardiac rehabilitation. These rates may seem small at an individual patient level. However, across an entire health care system the magnitude of this effect could affect thousands of patients who have long drive times and are not receiving health care services.

### Policy and Programmatic Implications

To mitigate access issues, including the potential impact of drive time, the VA has adopted multiple strategies, including expanding clinical sites into rural areas, offering telehealth options, allowing patients to receive care from local non-VA facilities, and providing transportation services, travel reimbursement, and overnight stays on VA property. However, despite all these strategies, drive time remains significantly associated with receipt of care.

Other options are available to address the impact of drive time. Although many physicians already try to consolidate appointments for patients with long drive times, most systems currently require physicians to identify this issue or patients to raise it. Alerts to physicians or administrative staff may increase the chances of consolidation of appointments. Health systems could also make spirometry available at more clinics or send mobile BMD testing to remote areas. To improve participation in cardiac rehabilitation, health systems could increase adoption of cardiac rehabilitation delivered via telehealth. Other considerations should include increasing telehealth capacity for other services including expanded reimbursement for telehealth services and investment in broadband infrastructure, bolstering the rural health care workforce to address medical staff shortages, and addressing paid sick leave policies to support patients and their caregivers who have to longer distances to receive care.^37^ Future research would be needed to quantify the effect of these potential interventions. Our findings may be of particular importance to clinicians who practice outside the VA, especially if they work in systems that have not adopted as many strategies to mitigate the impact of drive time as the VA already has.

### Limitations

Our study has several important limitations. First, we studied drive time, which assumes patients use private vehicles to travel to care. However, some patients use public transportation, which may take longer or shorter.^38,39^ Thus, travel time that accounts for mode of transportation would be a better measure of access to care. However, drive time is more easily captured and can be calculated using administrative data, while capturing travel time data would require surveying patients about modes of transportation and time required. Our findings suggest that drive time is a meaningful measure of travel burden. Second, we could not quantify drive time to services provided in non-VA settings. However, most services captured in our data were provided in a VA facility except for cardiac rehabilitation. Third, we have data on services received from the VA, VA-purchased care, or Medicare fee-for-service, but some patients who use the VA may also receive care through MA or private insurance^40^ and we did not have access to that data. However, the US Centers for Medicare and Medicaid Services reported that rural patients enrolled in MA received lower-quality care than their urban counterparts overall and specifically were less likely to receive BMD measurement for osteoporosis management and spirometry to confirm COPD diagnosis.^41^ There is no prior data addressing the association of drive times with receipt of services for privately insured patients; however, rural patients are less likely to have private insurance and generally have longer drive times to access care than urban patients.^42^ Addition of MA or private insurance data may alter the findings; this could be addressed in future studies. Finally, our study period predates the COVID-19 pandemic; there has been increased utilization of telehealth services within the VA. It is unclear how this has impacted care in general, but services like BMD measurement and spirometry cannot be performed by telehealth. Cardiac rehabilitation services can be provided by telehealth, but this remains uncommon.

## Conclusions

In this retrospective cohort study, longer drive time was associated with less frequent receipt of guideline-recommended services across multiple components of care. To improve quality of care and health outcomes, health systems and clinicians should adopt strategies to mitigate travel burden, even for urban patients.

## Notes

Supplement. **eTable 1.** Diagnosis and Procedure Codes Used to Define the Patients Eligible for Each Recommended Service **eTable 2.** Characteristics of Patients by Recommended Service **eTable 3.** Characteristics of Patients Eligible for Each Recommended Service, Stratified by Rurality **eTable 4.** Characteristics of the Women Eligible for Osteoporosis Screening by Drive Time to the Closest Osteoporosis Screening (N=110 780) **eTable 5.** Characteristics of the Patients Eligible for Spirometry by Drive Time to the Closest Spirometry (N=281 597) **eTable 6.** Characteristics of the Patients Eligible for Cardiac Rehabilitation by Drive Time to the Closest Cardiac Rehabilitation (N=73 146) **eTable 7.** Logistic Regression Analyses for Receipt of Recommended Services by Drive Time to the Closest Service among Patients ≥65 Years and Enrolled in Medicare Fee-For-Service **eTable 8.** Logistic Regression Analyses for Receipt of Recommended Services by Drive Time to the Closest Service Excluding Patients with Medicare Advantage
