# Supplementary material for: Drive Time and Receipt of Guideline-Recommended Screening, Diagnosis, and Treatment
Source: JAMA Netw Open. 2022 Nov 4;5(11):e2240290. doi: 10.1001/jamanetworkopen.2022.40290 (PMC9636523; doi:10.1001/jamanetworkopen.2022.40290)
Supplement: Supplement. — eTable 1. Diagnosis and Procedure Codes Used to Define the Patients Eligible for Each Recommended Service eTable 2. Characteristics of Patients by Recommended Service eTable 3. Characteristics of Patients Eligible for Each Recommended Service, Stratified by Rurality eTable 4. Characteristics of the Women Eligible for Osteoporosis Screening by Drive Time to the Closest Osteoporosis Screening (N=110 780) eTable 5. Characteristics of the Patients Eligible for Spirometry by Drive Time to the Closest Spirometry (N=281 597) eTable 6. Characteristics of the Patients Eligible for Cardiac Rehabilitation by Drive Time to the Closest Cardiac Rehabilitation (N=73 146) eTable 7. Logistic Regression Analyses for Receipt of Recommended Services by Drive Time to the Closest Service among Patients ≥65 Years and Enrolled in Medicare Fee-For-Service eTable 8. Logistic Regression Analyses for Receipt of Recommended Services by Drive Time to the Closest Service Excluding Patients with Medicare Advantage [file jamanetwopen-e2240290-s001.pdf]

# Supplemental Online Content

Baldomero AK, Kunisaki KM, Wendt CH, et al. Drive time and receipt of guideline-recommended screening, diagnosis, and treatment. *JAMA Netw Open.* 2022;5(11):e2240290. doi:10.1001/jamanetworkopen.2022.40290

**eTable 1.** Diagnosis and Procedure Codes Used to Define the Patients Eligible for Each Recommended Service

**eTable 2.** Characteristics of Patients by Recommended Service

**eTable 3.** Characteristics of Patients Eligible for Each Recommended Service, Stratified by Rurality

**eTable 4.** Characteristics of the Women Eligible for Osteoporosis Screening by Drive Time to the Closest Osteoporosis Screening (N=110 780)

**eTable 5.** Characteristics of the Patients Eligible for Spirometry by Drive Time to the Closest Spirometry (N=281 597)

**eTable 6.** Characteristics of the Patients Eligible for Cardiac Rehabilitation by Drive Time to the Closest Cardiac Rehabilitation (N=73 146)

**eTable 7.** Logistic Regression Analyses for Receipt of Recommended Services by Drive Time to the Closest Service among Patients ≥65 Years and Enrolled in Medicare Fee-For-Service

**eTable 8.** Logistic Regression Analyses for Receipt of Recommended Services by Drive Time to the Closest Service Excluding Patients with Medicare Advantage

This supplemental material has been provided by the authors to give readers additional information about their work.

© 2022 Baldomero AK et al. *JAMA Network Open.*

**eTable 1. Diagnosis and Procedure Codes used to Define the Patients Eligible for Each Recommended Service**

| Recommended Service | Excluded from the Analyses | Index Date | Outcome |
|---|---|---|---|
| **Osteoporosis screening among women ≥65 years** | | | |
| Women who were ≥65 years between 1/1/2016 to 12/31/2019 | Any of the following diagnoses or medications 12 months prior to index date.[1]<br><br>• Osteoporosis diagnoses<br>ICD-9 733.0X<br>ICD-10 M80.X, M81.X<br>• Osteoporosis medication prescriptions (≥1 outpatient prescription for alendronate, calcitonin, denosumab, ibandronate, raloxifene, risedronate, teriparatide, or zoledronic acid)<br>• Prior history of qualifying hip fracture<br>ICD-9 744.14, 820.0X, 920.2X<br>ICD10 M84.459X, S72.019A, S72.033A S72.036A, S72.109A, S72.143A, S72.146A, S72.23XA, S72.26XA, S72.009A<br>• Evidence of underlying conditions known to impact bone health:<br>End-stage renal disease: ICD-9 585.6; ICD-10 N18.6<br>Bone metastases: ICD-9 198.5; ICD-10 C79.5X<br>Cushing syndrome: ICD-9 255.0; ICD-10 E24.X<br>Paget disease: ICD-9 731.0; ICD-10 M88.9<br>Osteogenesis imperfecta: ICD-9 756.51; ICD-10 Q78.0<br>Malignant neoplasm of long bones: ICD-9 170.7; ICD-10 C40.20 | We defined the index date as the date when the woman turned 65 years old. | Bone measurement density (BMD) testing by dual-energy x-ray absorptiometry (DXA) of central/peripheral skeleton or quantitative ultrasound (QUS) bone density measurement within two years of the index date.[1]<br><br>CPT codes: 76070, 76071, 76075, 76076, 76077, 76078, 76977, 77078, 77079, 77080, 77081, 77082, 77083, 77085, 78350, 78351<br>HCPCS code: G0130 |
| **Spirometry for confirmation of COPD diagnosis** | | | |
| Patients with diagnosis code for COPD between 1/1/2016 to 12/31/2019[2]:<br>ICD-10: J40, J41.x, J42, J43.x, J44 | We excluded patients with:<br>• Less than two ICD diagnosis codes for COPD, and<br>• Not newly-diagnosed with COPD between 2016 and 2019 | We defined the index date as the date of the first diagnosis code for COPD. | Spirometry within two years before or after the index date defined by:<br><br>CPT codes: 94010, 94014, 94015, 94016, 94060, 94070, 94375, 95070, 94620, 95071; or Pulmonary Function Test clinic stop code 104 |

© 2022 Baldomero AK et al. *JAMA Network Open.*

| eTable 1. Diagnosis and Procedure Codes used to Define the Patients Eligible for Each Recommended Service | | | |
|---|---|---|---|
| **Recommended Service** | **Excluded from the Analyses** | **Index Date** | **Outcome** |
| **Cardiac rehabilitation after hospitalization for an acute MI, PCI, or CABG surgery** | | | |
| Hospital discharge diagnosis or procedure codes for AMI, PCI, or CABG between 1/1/2016 and 12/31/2019.[3,4]<br><br>Acute myocardial infarction (MI):<br>ICD-10: I21.x-I23.x, and I24.x<br><br>Percutaneous coronary intervention (PCI):<br>ICD-10 codes: 02703ZZ, 02704ZZ, 02713ZZ, 02714ZZ, 02723ZZ, 02724ZZ, 02733ZZ, 02734ZZ, 02C03ZZ, 02700ZZ, 02710ZZ, 02720ZZ, 02730ZZ, 02C00ZZ, 02C10ZZ, 02C30ZZ, 02C20ZZ;<br>CPT codes: codes 92973, 92974, 92980-92982, 92984, 92995, 92996, 92921, 92924, 92925, 92928, 92929, 92933, 92934, 92937, 92938, 92943, 92944;<br>HCPCS codes: G0290 and G0291<br><br>Coronary artery bypass (CABG):<br>ICD-10 codes: 0210093, 02100A3, 02100J3, 02100K3, 02100Z3, 0210493, 02104A3, 02104J3, 02104K3, 02104Z3;<br>CPT codes: 33510-33514, 33516-33519, 33521-33523, 33530, 33533-33536, 33572, 35600, and 93564;<br>HCPCS codes: S2205-S2209<br><br>Note: Among patients with multiple hospital discharge diagnosis/procedure codes, the first one was selected. Patients who died within 30 days of the index date were excluded in the analyses. | Patients who died within 30 days of discharge were excluded from the analysis. | We defined the index date as the initial hospital discharge date between 1/1/2016 and 12/31/2019. | Cardiac rehabilitation within 12 months after index date was defined by[3]:<br><br>CPT codes: 93797, 93798; or HCPCS codes: S9472, S9473, G0422, G0423; or Cardiopulmonary rehabilitation program clinic stop code 231 |

© 2022 Baldomero AK et al. *JAMA Network Open.*

| eTable 2. Characteristics of Patients by Recommended Service | | | |
|---|---|---|---|
| | **Osteoporosis Screening (N=110,780)** | **Spirometry (N=281,130)** | **Cardiac Rehabilitation (N=73,146)** |
| **Age,** years, mean±SD | 66.7±5.4 | 68.2±11.5 | 70.0±10.8 |
| **Male,** No. (%) | - | 268,999 (95.7) | 71,217 (97.4) |
| **Race/Ethnicity,** No. (%) | | | |
| American Indian/Alaska Native | 1,181 (1.1) | 2,967 (1.1) | 676 (0.9) |
| Asian | 672 (0.6) | 1,390 (0.5) | 489 (0.7) |
| Black/African American | 19,421 (17.5) | 37,834 (13.5) | 15,213 (20.8) |
| Native Hawaiian/Pacific Islander | 965 (0.9) | 2,134 (0.8) | 580 (0.8) |
| White | 63,403 (57.2) | 217,608 (77.4) | 52,144 (71.3) |
| Unknown/Declined | 25,138 (22.7) | 19,197 (6.8) | 4,044 (5.5) |
| **Area Deprivation Index[a]** (percentile), mean±SD | 54.3±25.4 | 58.5±24.8 | 57.9±26.0 |
| **Charlson Comorbidity Index[b],** No. (%) | | | |
| 0 | 90,116 (81.4) | 87,564 (31.2) | 11,729 (16.0) |
| 1-2 | 16,617 (15.0) | 125,985 (44.8) | 34,755 (47.5) |
| ≥3 | 4,047 (3.7) | 67,581 (24.0) | 26,662 (36.5) |
| **Geographic Region[c],** No. (%) | | | |
| Midwest | 15,075 (14.9) | 58,954 (22.8) | 13,816 (20.5) |
| Northeast | 12,428 (12.3) | 38,632 (15.0) | 8.754 (13.0) |
| South | 45,169 (44.7) | 105,741 (41.0) | 28,495 (42.3) |
| West | 28,478 (28.2) | 54,915 (21.3) | 16,322 (24.2) |
| **Rurality,** No. (%) | | | |
| Urban | 75,921 (68.5) | 169,800 (60.4) | 49,725 (68.0) |
| Rural | 34,859 (31.5) | 111,330 (39.6) | 23,421 (32.0) |
| **Drive Time to the Closest Service,** No. (%) | | | |
| ≤30 minutes | 49,377 (44.6) | 147,548 (52.5) | 34,419 (47.1) |
| 31-60 minutes | 25,337 (22.9) | 73,738 (26.2) | 16,112 (22.0) |
| 61-90 minutes | 13,453 (12.1) | 32,142 (11.4) | 7,718 (10.6) |
| 91-120 minutes | 9,684 (8.7) | 16,138 (5.7) | 6,353 (8.7) |
| >120 minutes | 12,929 (11.7) | 11,564 (4.1) | 8,544 (11.7) |

[a]Area Deprivation Index provides percentile ranking of neighborhoods by census block groups based on the aggregated domains of income, education, employment, and housing quality (percentile ranged from 1 to 100, with higher scores indicating higher levels of socioeconomic disadvantage).[5]

[b]Charlson Comorbidity Index scores range from 0 to 33, with higher scores indicating greater disease burden and increased risk of death within 1 year.[6]

[c]Geographic regions were divided into four categories according to each patient's Veterans Integrated Services Networks (VISN) which are regional systems of care working together to meet local health care needs and provides access to care. Midwest includes patients from VISNs 10, 15, 17, and 23; Northeast from VISNs 1, 2, and 4; South from VISNs 6, 7, 8, 9, 16, and 17; and West from VISNs 19, 20, 21, and 22. Numbers may not sum to group totals as some patients were assigned to networks outside of the specified VISNs.

© 2022 Baldomero AK et al. *JAMA Network Open.*

| eTable 3. Characteristics of Patients Eligible for Each Recommended Service, Stratified by Rurality | | | | | | |
|---|---|---|---|---|---|---|
| | Osteoporosis Screening (N=110,780) | | Spirometry (N=281,263) | | Cardiac Rehabilitation (N=73,146) | |
| | Urban (n=75,921) | Rural (n=34,859) | Urban (n=169,800) | Rural (n=111,330) | Urban (n=49,725) | Rural (n=23,421) |
| **Age,** years, mean±SD | 66.6±5.4 | 67.0±5.5 | 68.2±11.8 | 68.2±11.1 | 69.9±11.1 | 70.2±9.9 |
| **Male** (%) | - | - | 161,699 (95.2) | 107,300 (96.4) | 48,267 (97.1) | 22,950 (98.0) |
| **Race/Ethnicity** (%) | | | | | | |
| American Indian/Alaska Native | 675 (0.9) | 506 (1.5) | 1,520 (0.9) | 1,447 (1.3) | 404 (0.8) | 272 (1.2) |
| Asian | 583 (0.8) | 89 (0.3) | 1,180 (0.7) | 210 (0.2) | 463 (0.9) | 26 (0.1) |
| Black/African American | 16,596 (21.9) | 2,825 (8.1) | 30,931 (18.2) | 6,903 (6.2) | 13,343 (26.8) | 1,870 (8.0) |
| Native Hawaiian/Pacific Islander | 702 (0.9) | 263 (0.8) | 1,481 (0.9) | 653 (0.6) | 447 (0.9) | 133 (0.6) |
| White | 39,595 (52.2) | 23,808 (68.3) | 122,936 (72.4) | 94,672 (85.0) | 32,204 (64.8) | 19,940 (85.1) |
| Unknown/Declined | 17,770 (23.4) | 7,368 (21.1) | 11,752 (6.9) | 7,445 (6.7) | 2,864 (5.8) | 1,180 (5.0) |
| **Area Deprivation Index**[a] (percentile), mean±SD | 50.8±25.9 | 62.0±22.4 | 54.2±26.1 | 65.0±21.1 | 54.1±27.3 | 65.9±20.9 |
| **Charlson Comorbidity Index**[b] (%) | | | | | | |
| 0 | 61,831 (81.4) | 28,285 (81.1) | 50,633 (29.8) | 36,931 (33.2) | 7,634 (15.4) | 4,095 (17.5) |
| 1-2 | 11,266 (14.8) | 5,351 (15.4) | 76,041 (44.8) | 49,944 (44.9) | 23,576 (47.4) | 11,179 (47.7) |
| ≥3 | 2,824 (3.7) | 1,223 (3.5) | 43,126 (25.4) | 24,455 (22.0) | 18,515 (37.2) | 8,147 (34.8) |
| **Geographic Region**[c] (%) | | | | | | |
| Midwest | 8,816 (12.8) | 6,259 (19.5) | 29,728 (19.0) | 29,226 (28.6) | 8,635 (18.9) | 5,181 (24.0) |
| Northeast | 8,656 (12.5) | 3,772 (11.7) | 26,194 (16.8) | 14,439 (12.2) | 6,536 (14.3) | 2,218 (10.3) |
| South | 31,568 (45.7) | 13,601 (42.3) | 64,628 (41.4) | 41,113 (40.3) | 18,133 (39.6) | 10,362 (48.0) |
| West | 19,979 (29.0) | 8,499 (26.5) | 35,555 (22.8) | 19,360 (19.0) | 12,485 (27.3) | 3,837 (17.8) |
| **Drive Time to the Closest Service,** minutes, median (interquartile range) | 28.0 (17.0-55.0) | 76.0 (46.0-118.0) | 20.0 (13.0-34.0) | 51.0 (31.0-80.0) | 23.0 (15.0-44.0) | 69.0 (42.0-112.0) |
| ≤30 minutes | 43,174 (56.9) | 6,203 (17.8) | 120,046 (70.7) | 27,502 (24.7) | 31,354 (63.1) | 3,065 (13.1) |
| 31-60 minutes | 16,779 (22.1) | 8,558 (24.6) | 34,308 (20.2) | 39,430 (35.4) | 8,948 (18.0) | 7,164 (30.6) |
| 61-90 minutes | 6,395 (8.4) | 7,058 (20.3) | 8,941 (5.3) | 23,201 (20.8) | 2,919 (5.9) | 4,799 (20.5) |
| 91-120 minutes | 4,395 (5.8) | 5,289 (15.2) | 3,917 (2.3) | 12,221 (11.0) | 5,965 (6.0) | 3,388 (14.5) |
| >120 minutes | 5,178 (6.8) | 7,751 (22.2) | 2,588 (1.5) | 8,976 (8.1) | 3,539 (7.1) | 5,005 (21.4) |

[a]Area Deprivation Index provides percentile ranking of neighborhoods by census block groups based on the aggregated domains of income, education, employment, and housing quality (percentile ranged from 1 to 100, with higher scores indicating higher levels of socioeconomic disadvantage).[5]
[b]Charlson Comorbidity Index scores range from 0 to 33, with higher scores indicating greater disease burden and increased risk of death within 1 year.[6]
[c]Geographic regions were divided into four categories according to each patient's Veterans Integrated Services Networks (VISN) which are regional systems of care working together to meet local health care needs and provides access to care. Midwest includes patients from VISNs 10, 15, 17, and 23; Northeast from VISNs 1, 2, and 4; South from VISNs 6, 7, 8, 9, 16, and 17; and West from VISNs 19, 20, 21, and 22. Numbers may not sum to group totals as some patients were assigned to networks outside of the specified VISNs.

© 2022 Baldomero AK et al. *JAMA Network Open.*

| **eTable 4. Characteristics of the Women Eligible for Osteoporosis Screening by Drive Time to the Closest Osteoporosis Screening (N=110,780)** | | | | | |
|---|---|---|---|---|---|
| | **≤30 minutes (n=49,377)** | **31-60 minutes (n=25,337)** | **61-90 minutes (n=13,453)** | **91-120 minutes (n=9,684)** | **>120 minutes (n=12,929)** |
| **Age,** years, mean±SD | 65.9±4.9 | 65.9±4.9 | 67.1±5.5 | 68.3±5.9 | 69.9±6.2 |
| **Race/Ethnicity** (%) | | | | | |
| American Indian/Alaska Native | 469 (1.0) | 256 (1.0) | 162 (1.2) | 101 (1.0) | 193 (1.5) |
| Asian | 440 (0.9) | 135 (0.5) | 42 (0.3) | 24 (0.3) | 31 (0.2) |
| Black/African American | 11,812 (23.9) | 4,006 (15.8) | 1,669 (12.4) | 1,233 (12.7) | 701 (5.4) |
| Native Hawaiian/Pacific Islander | 467 (1.0) | 219 (0.9) | 103 (0.8) | 81 (0.8) | 95 (0.7) |
| White | 23,972 (48,6) | 15,487 (61.1) | 8,792 (65.4) | 6,235 (64.4) | 8,917 (69.0) |
| Unknown/Declined | 12,217 (24.7) | 5,234 (20.8) | 2,685 (20.0) | 2,010 (20.8) | 2,992 (23.1) |
| **Area Deprivation Index**[a] (percentile), mean±SD | 50.3±26.1 | 51.4±25.1 | 61.3±23.6 | 62.9±22.9 | 60.3±22.6 |
| **Charlson Comorbidity Index**[b] (%) | | | | | |
| 0 | 40,222 (81.5) | 20,624 (81.4) | 10,803 (80.3) | 7,844 (81.0) | 10,623 (82.2) |
| 1-2 | 7,179 (14.5) | 3,802 (15.0) | 2,163 (16.1) | 1,537 (15.9) | 1,936 (15.0) |
| ≥3 | 1,976 (4.0) | 911 (3.6) | 487 (3.6) | 303 (3.1) | 370 (2.9) |
| **Geographic Region**[c] (%) | | | | | |
| Midwest | 6,404 (14.2) | 3,219 (14.2) | 2.030 (16.3) | 1,414 (15.9) | 2,008 (16.7) |
| Northeast | 5,047 (11.2) | 3,627 (16.0) | 1,828 (14.7) | 909 (10.2) | 1,017 (8.4) |
| South | 19,745 (43.8) | 10,519 (46.3) | 5,951 (47.9) | 4,557 (51.2) | 4,397 (36.5) |
| West | 13,840 (30.7) | 5,364 (23.6) | 2,615 (21.1) | 2,018 (22.7) | 4,641 (38.5) |

[a]Area Deprivation Index provides percentile ranking of neighborhoods by census block groups based on the aggregated domains of income, education, employment, and housing quality (percentile ranged from 1 to 100, with higher scores indicating higher levels of socioeconomic disadvantage).[5]
[b]Charlson Comorbidity Index scores range from 0 to 33, with higher scores indicating greater disease burden and increased risk of death within 1 year.[6]
[c]Geographic regions were divided into four categories according to each patient's Veterans Integrated Services Networks (VISN) which are regional systems of care working together to meet local health care needs and provides access to care. Midwest includes patients from VISNs 10, 15, 17, and 23; Northeast from VISNs 1, 2, and 4; South from VISNs 6, 7, 8, 9, 16, and 17; and West from VISNs 19, 20, 21, and 22. Numbers may not sum to group totals as some patients are assigned to networks outside of the specified VISNs.

© 2022 Baldomero AK et al. *JAMA Network Open.*

| eTable 5. Characteristics of the Patients Eligible for Spirometry by Drive Time to the Closest Spirometry (N=281,597) | | | | | |
|---|---|---|---|---|---|
| | **≤30 minutes (n=147,548)** | **31-60 minutes (n=73,738)** | **61-90 minutes (n=32,142)** | **91-120 minutes (n=16,138)** | **>120 minutes (n=11,564)** |
| **Age,** years, mean±SD | 68.1±11.9 | 68.4±11.3 | 68.3±11.0 | 68.0±11.1 | 68.3±11.2 |
| **Male,** No. (%) | 140,598 (95.3) | 70,799 (96.0) | 30,960 (96.3) | 15,531 (96.2) | 11,111 (96.1) |
| **Race/ethnicity,** No. (%) | | | | | |
| American Indian/Alaska Native | 1,444 (1.0) | 784 (1.1) | 323 (1.0) | 181 (1.1) | 235 (2.0) |
| Asian | 1,085 (0.7) | 216 (0.3) | 59 (0.2) | 14 (0.1) | 16 (0.1) |
| Black/African American | 26,120 (17.7) | 6,739 (9.1) | 2,746 (8.5) | 1,614 (10.0) | 615 (5.3) |
| Native Hawaiian/Pacific Islander | 1,343 (0.9) | 479 (0.7) | 159 (0.5) | 98 (0.6) | 55 (0.5) |
| White | 107,654 (73.0) | 60,723 (82.4) | 26,617 (82.8) | 13,047 (80.9) | 9,567 (82.7) |
| Unknown/Declined | 9,902 (6.7) | 4,797 (6.5) | 2,238 (7.0) | 1,184 (7.3) | 1,076 (9.3) |
| **Area Deprivation Index**[a] (percentile), mean±SD | 55.3±25.8 | 58.3±23.9 | 66.1±21.6 | 68.8±20.3 | 64.1±22.2 |
| **Charlson Comorbidity Index**[b]**,** No. (%) | | | | | |
| 0 | 42,380 (28.7) | 23,998 (32.5) | 11,169 (34.7) | 5,604 (34.7) | 4,413 (38.2) |
| 1-2 | 66,980 (45.4) | 32,732 (44.4) | 14,003 (43.6) | 7,228 (44.8) | 5,042 (43.6) |
| ≥3 | 38,188 (25.9) | 17,008 (23.1) | 6,970 (21.7) | 6,970 (20.5) | 2,109 (18.2) |
| **Geographic Region**[c]**,** No. (%) | | | | | |
| Midwest | 32,557 (23.8) | 14,930 (22.5) | 6,366 (21.8) | 3,080 (21.0) | 2,021 (18.4) |
| Northeast | 20,438 (14.9) | 11,311 (17.0) | 4,870 (16.7) | 1,258 (8.6) | 755 (6.9) |
| South | 50,553 (36.9) | 28,710 (43.2) | 13,526 (46.4) | 8,062 (55.1) | 4,890 (44.6) |
| West | 33,430 (24.4) | 11,565 (17.4) | 4,380 (15.0) | 2,238 (15.3) | 3,302 (30.1) |

[a]Area Deprivation Index provides percentile ranking of neighborhoods by census block groups based on the aggregated domains of income, education, employment, and housing quality (percentile ranged from 1 to 100, with higher scores indicating higher levels of socioeconomic disadvantage).[5]
[b]Charlson Comorbidity Index scores range from 0 to 33, with higher scores indicating greater disease burden and increased risk of death within 1 year.[6]
[c]Geographic regions were divided into four categories according to each patient's Veterans Integrated Services Networks (VISN) which are regional systems of care working together to meet local health care needs and provides access to care. Midwest includes patients from VISNs 10, 15, 17, and 23; Northeast from VISNs 1, 2, and 4; South from VISNs 6, 7, 8, 9, 16, and 17; and West from VISNs 19, 20, 21, and 22. Numbers may not sum to group totals as some patients are assigned to networks outside of the specified VISNs.

© 2022 Baldomero AK et al. *JAMA Network Open.*

| **eTable 6. Characteristics of the Patients Eligible for Cardiac Rehabilitation by Drive Time to the Closest Cardiac Rehabilitation (N=73,146)** | | | | | |
|---|---|---|---|---|---|
| | **≤30 minutes (n=34,419)** | **31-60 minutes (n=16,112)** | **61-90 minutes (n=7,718)** | **91-120 minutes (n=6,353)** | **>120 minutes (n=8,544)** |
| **Age,** years, mean±SD | 70.2±11.3 | 69.6±10.4 | 69.7±10.0 | 69.7±10.3 | 70.1±10.3 |
| **Male** (%) | 33,353 (96.9) | 15,761 (97.2) | 7,540 (97.7) | 6,209 (97.7) | 8,354 (97.8) |
| **Race/Ethnicity** (%) | | | | | |
| American Indian/Alaska Native | 295 (0.9) | 121 (0.8) | 73 (1.0) | 61 (1.0) | 126 (1.5) |
| Asian | 340 (1.0) | 89 (0.6) | 14 (0.2) | 25 (0.4) | 21 (0.3) |
| Black/African American | 9,657 (28.1) | 2,505 (15.6) | 1,043 (13.5) | 1,094 (17.2) | 914 (10.7) |
| Native Hawaiian/Pacific Islander | 301 (0.9) | 111 (0.7) | 65 (0.8) | 36 (0.6) | 67 (0.8) |
| White | 21,872 (63.6) | 12,421 (77.1) | 6,137 (79.5) | 2,846 (76.3) | 6,868 (80.4) |
| Unknown/Declined | 1,954 (5.6) | 865 (5.4) | 386 (5.0) | 291 (4.6) | 548 (6.4) |
| **Area Deprivation Index**[a] (percentile), mean±SD | 53.2±27.8 | 57.5±24.6 | 65.8±22.0 | 64.6±22.4 | 65.1±22.7 |
| **Charlson Comorbidity Index**[b] (%) | | | | | |
| 0 | 4,981 (14.5) | 2,545 (15.7) | 1,309 (17.0) | 1,207 (19.0) | 1,698 (19.9) |
| 1-2 | 16,492 (47.9) | 7,600 (47.2) | 3,679 (47.7) | 2,959 (46.6) | 4,025 (47.1) |
| ≥3 | 12,946 (37.6) | 5,977 (37.1) | 2,731 (35.4) | 2,187 (34.4) | 2,821 (33.0) |
| **Geographic Region**[c] (%) | | | | | |
| Midwest | 6,554 (20.6) | 3,362 (22.8) | 1,424 (19.6) | 953 (16.3) | 1,523 (19.6) |
| Northeast | 4,350 (13.4) | 1,816 (12.3) | 733 (10.1) | 1,077 (18.5) | 878 (11.3) |
| South | 11,928 (37.6) | 6,848 (46.5) | 4,140 (56.8) | 2,710 (46.5) | 2,869 (36.9) |
| West | 9,033 (28.4) | 2,701 (18.3) | 988 (13.6) | 1,092 (18.7) | 2,508 (32.2) |

[a]Area Deprivation Index provides percentile ranking of neighborhoods by census block groups based on the aggregated domains of income, education, employment, and housing quality (percentile ranged from 1 to 100, with higher scores indicating higher levels of socioeconomic disadvantage).[5]
[b]Charlson Comorbidity Index scores range from 0 to 33, with higher scores indicating greater disease burden and increased risk of death within 1 year.[6]
[c]Geographic regions were divided into four categories according to each patient's Veterans Integrated Services Networks (VISN) which are regional systems of care working together to meet local health care needs and provides access to care. Midwest includes patients from VISNs 10, 15, 17, and 23; Northeast from VISNs 1, 2, and 4; South from VISNs 6, 7, 8, 9, 16, and 17; and West from VISNs 19, 20, 21, and 22. Numbers may not sum to group totals as some patients are assigned to networks outside of the specified VISNs.

© 2022 Baldomero AK et al. *JAMA Network Open.*

| eTable 7. Logistic Regression Analyses for Receipt of Recommended Services by Drive Time to the Closest Service among Patients ≥65 Years and Enrolled in Medicare Fee-For-Service[a] | | | | |
|---|---|---|---|---|
| | **Patients, No.** | **Adjusted Rates, %** | **Adjusted Odds Ratio** | **P Value for** |
| | | **(95% Confidence Interval)** | | **Linear Trend** |
| **Osteoporosis Screening** | 93,052 | | | |
| ≤30 minutes | 38,339 | 38.1 (37.6-38.7) | 1.00 | 0.32 |
| 31-60 minutes | 22,361 | 37.1 (36.4-37.8) | 0.96 (0.92-0.99) | |
| 61-90 minutes | 11,996 | 35.2 (34.3-36.2) | 0.88 (0.84-0.93) | |
| 91-120 minutes | 8,600 | 36.0 (34.9-37.1) | 0.91 (0.87-0.96) | |
| >120 minutes | 11,756 | 36.2 (35.3-37.2) | 0.92 (0.88-0.97) | |
| **Spirometry** | 113,099 | | | |
| ≤30 minutes | 56,634 | 58.5 (58.1-58.9) | 1.00 | <0.001 |
| 31-60 minutes | 30,900 | 58.4 (57.9-59.0) | 1.00 (0.97-1.03) | |
| 61-90 minutes | 13,707 | 55.7 (54.8-56.5) | 0.89 (0.85-0.93) | |
| 91-120 minutes | 6,827 | 55.1 (53.9-56.3) | 0.87 (0.83-0.92) | |
| >120 minutes | 5,031 | 53.3 (51.9-54.7) | 0.81 (0.76-0.86) | |
| **Cardiac Rehabilitation** | 42,196 | | | |
| ≤30 minutes | 19,159 | 16.4 (15.8-17.0) | 1.00 | <0.001 |
| 31-60 minutes | 9,521 | 16.7 (15.9-17.4) | 1.02 (0.95-1.09) | |
| 61-90 minutes | 4,677 | 15.9 (15.9-14.9) | 0.97 (0.88-1.06) | |
| 91-120 minutes | 3,729 | 13.4 (12.3-14.5) | 0.79 (0.71-0.87) | |
| >120 minutes | 5,110 | 13.9 (13.0-14.9) | 0.83 (0.75-0.90) | |

[a]We included patients in the sensitivity analyses if they were enrolled in Medicare Fee-For-Service during the ascertainment period. Models adjusted for age, sex, race, urban/rural address, Charlson Comorbidity Index, and Area Deprivation Index. The omnibus likelihood-ratio chi-square test to assess whether drive time was associated with receipt of services is p<0.001 for all models.

© 2022 Baldomero AK et al. *JAMA Network Open.*

| eTable 8. Logistic Regression Analyses for Receipt of Recommended Services by Drive Time to the Closest Service Excluding Patients with Medicare Advantage[a] | | | | |
|---|---|---|---|---|
| | Patients, No. | Adjusted Rates, % (95% Confidence Interval) | Adjusted Odds Ratio (95% Confidence Interval) | P Value for Linear Trend |
| **Osteoporosis Screening** | 91,879 | | | |
| ≤30 minutes | 40,996 | 37.1 (36.5-37.6) | 1.00 | 0.07 |
| 31-60 minutes | 20,462 | 37.4 (36.7-38.1) | 1.01 (0.98-1.05) | |
| 61-90 minutes | 11,086 | 35.6 (34.6-36.5) | 0.94 (0.89-0.99) | |
| 91-120 minutes | 8,124 | 36.1 (34.9-37.2) | 0.96 (0.91-1.01) | |
| >120 minutes | 11,211 | 36.0 (35.0-37.0) | 0.96 (0.91-1.01) | |
| **Spirometry** | 219,799 | | | |
| ≤30 minutes | 114,506 | 54.4 (54.1-54.7) | 1.00 | <0.001 |
| 31-60 minutes | 56,896 | 55.3 (54.9-55.7) | 1.04 (1.02-1.06) | |
| 61-90 minutes | 25,621 | 52.2 (51.6-52.8) | 0.92 (0.89-0.94) | |
| 91-120 minutes | 13,090 | 51.0 (50.1-51.8) | 0.87 (0.84-0.91) | |
| >120 minutes | 9,686 | 49.7 (48.6-50.7) | 0.83 (0.79-0.87) | |
| **Cardiac Rehabilitation** | 60,920 | | | |
| ≤30 minutes | 28,232 | 15.0 (14.6-15.5) | 1.00 | <0.001 |
| 31-60 minutes | 13,379 | 15.6 (15.0-16.2) | 1.04 (0.98-1.11) | |
| 61-90 minutes | 6,526 | 14.5 (13.7-15.4) | 0.96 (0.88-1.04) | |
| 91-120 minutes | 5,371 | 12.3 (11.5-13.2) | 0.80 (0.73-0.87) | |
| >120 minutes | 7,412 | 11.8 (11.1-12.6) | 0.76 (0.70-0.82) | |

[a]We excluded patients in the sensitivity analyses if they were enrolled in Medicare Advantage during the ascertainment period. Models adjusted for age, sex, race, urban/rural address, Charlson Comorbidity Index, and Area Deprivation Index. The omnibus likelihood-ratio chi-square test to assess whether drive time was associated with receipt of services is p<0.001 for spirometry and cardiac rehabilitation; p=0.02 for osteoporosis screening.

© 2022 Baldomero AK et al. *JAMA Network Open.*

## References


1. Gillespie CW, Morin PE. Trends and Disparities in Osteoporosis Screening Among Women in the United States, 2008-2014. *The American Journal of Medicine*. 2017 Mar 2017;130(3)doi:10.1016/j.amjmed.2016.10.018
2. Gothe H, Rajsic S, Vukicevic D, et al. Algorithms to identify COPD in health systems with and without access to ICD coding: a systematic review. OriginalPaper. *BMC Health Services Research*. 2019-10-22 2019;19(1):1-24. doi:doi:10.1186/s12913-019-4574-3
3. Krishnamurthi N, Schopfer DW, Shen H, Whooley MA. Association of Cardiac Rehabilitation With Survival Among US Veterans. *JAMA Network Open*. 2021;3(3)doi:10.1001/jamanetworkopen.2020.1396
4. Derington C, Heath L, Kao D, Delate T. Validation of algorithms to identify elective percutaneous coronary interventions in administrative databases. *PloS one*. 04/07/2020 2020;15(4)doi:10.1371/journal.pone.0231100
5. Kind A, Buckingham W. Making Neighborhood-Disadvantage Metrics Accessible - The Neighborhood Atlas. *N Engl J Med*. Jun 2018;378(26):2456-2458. doi:10.1056/NEJMp1802313
6. Quan H, Sundararajan V, Halfon P, et al. Coding algorithms for defining comorbidities in ICD-9-CM and ICD-10 administrative data. *Medical Care*. 2005 Nov 2005;43(11):1130-1139. doi:10.1097/01.mlr.0000182534.19832.83


© 2022 Baldomero AK et al. *JAMA Network Open.*
